# The New Comorbidity Index for Predicting Survival in Elderly Dialysis Patients: A Long-Term Population-Based Study

**DOI:** 10.1371/journal.pone.0068748

**Published:** 2013-08-06

**Authors:** Wei-Chih Kan, Jhi-Joung Wang, Shuo-Yu Wang, Yih-Min Sun, Chien-Ya Hung, Chin-Chen Chu, Chin-Li Lu, Shih-Feng Weng, Chung-Ching Chio, Chih-Chiang Chien

**Affiliations:** 1 Department of Nephrology, Chi-Mei Medical Center, Tainan, Taiwan; 2 Department of Medical Laboratory Science and Biotechnology, Chung Hwa University of Medical Technology, Tainan, Taiwan; 3 Southern Taiwan University, Tainan, Taiwan; 4 Department of Medical Research Chi-Mei Medical Center, Tainan, Taiwan; 5 Department of Pediatrics, Chi-Mei Medical Center, Tainan, Taiwan; 6 Department of Occupational Safety and Health, Chung Hwa University of Medical Technology, Tainan, Taiwan; 7 Department of Food Nutrition, Chung Hwa University of Medical Technology, Tainan, Taiwan; 8 Department of Neurological Surgery, Chi-Mei Medical Center, Tainan, Taiwan; Queen's University Belfast, United Kingdom

## Abstract

**Background:**

The worldwide elderly (≥65 years old) dialysis population has grown significantly. This population is expected to have more comorbid conditions and shorter life expectancies than the general elderly population. Predicting outcomes for this population is important for decision-making. Recently, a new comorbidity index (nCI) with good predictive value for patient outcomes was developed and validated in chronic dialysis patients regardless of age. Our study examined the nCI outcome predictability in elderly dialysis patients.

**Methods and Findings:**

For this population-based cohort study, we used Taiwan's National Health Insurance Research Database of enrolled elderly patients, who began maintenance dialysis between January 1999 and December 2005. A total of 21,043 incident dialysis patients were divided into 4 groups by nCI score (intervals ≤3, 4–6, 7–9, ≥10) and followed nearly for 10 years. All-cause mortality and life expectancy were analyzed. During the follow-up period, 11272 (53.55%) patients died. Kaplan-Meier curves showed significant group difference in survival (log-rank: *P*<0.001). After stratification by age, life expectancy was found to be significantly longer in groups with lower nCI scores.

**Conclusion:**

The nCI, even without the age component, is a strong predictor of mortality in elderly dialysis patients. Because patients with lower nCI scores may predict better survival, more attention should paid to adequate dialysis rather than palliative care, especially in those without obvious functional impairments.

## Introduction

The worldwide dialysis population is growing considerably, and its mortality rate is much higher than that of the general population [1]. Dialysis patients also have a high prevalence of comorbidities, including atherosclerotic cardiovascular disease (ACVD), congestive heart failure (CHF), hypertension (HTN), diabetes mellitus (DM), and cognitive and functional impairment, which in itself is often one of the risk factors for mortality [1], [2]. In addition, age per se is a strong predictor of mortality in many studies [3], [4]. Because the number of elderly people has increased considerably in many countries over the past decade, the elderly dialysis population has likewise grown significantly [1]. Thus far, elderly (≥65 years old) patients have been the most rapidly rising age group in the dialysis population in many developed and developing countries [1]. Because this population has always been known to have multiple comorbid illnesses and a higher mortality rate than the general population, palliative care has always been considered the primary means of treating them [5]. Several comorbidity index scoring systems have been used to objectively evaluate the prognosis of these patients. These indexes include the Charlson comorbidity index (CCI), index of co-existent diseases (ICED), Wright-Khan indexes, and the Davies et al. index [6]–[9]. Among these index systems, the CCI has been widely used in many longitudinal studies of patients with a variety of diseases. Thus, some studies have introduced the CCI and examined its validity in end-stage renal disease (ESRD) patients [10], in peritoneal dialysis patients [11], and in maintenance hemodialysis (MHD) patients [12]. Khan et al. designed a comorbidity index for survival analysis with a study population of 375 dialysis patients [8], and Davies et al. used another comorbidity index to analyze 97 continuous ambulatory peritoneal dialysis patients [9]. The CCI was significantly more predictive of mortality in dialysis patients than were these other two instruments [10], [11]. Recently, Liu et al. modified the CCI and developed a new comorbidity index (nCI) for dialysis patients, which was found to be a better predictor than the CCI [13]. The nCI covers comorbid conditions but not the age, one of the components of the original CCI. In the present study, using data obtained from the Taiwan National Health Insurance Research Database (NHIRD) [14], we investigated the predictive value of the nCI in elderly dialysis patients. We hypothesized that the nCI would be both useful and valid as a predictor of mortality in elderly dialysis patients.

## Methods

### Database

Taiwan has provided almost all of its residents with a compulsory universal National Health Insurance (NHI) since 1995 [14]. Except for prison inmates, almost all residents are enrolled. Under this program, all medical institutions must use standard computerized claim documents for medical expenses. Patients with end-stage renal disease (ESRD) are eligible for every type of renal replacement therapy without any charge, and all their expenses are covered by NHI.

The NHIRD contains nearly all (99%) inpatient and outpatient medical benefit claims for the 23 million residents of Taiwan, and has been used extensively in various studies [14]. This database provides a great deal of information, including gender, birth date, dates of admission and discharge, the medical institutions providing the services, the ICD-9-CM (International Classification of Diseases, 9th Revision, Clinical Modification) diagnostic and procedure codes (up to five each), and encrypted outcomes. In this study, we retrieved ambulatory care claims, all inpatient claims, and the updated registry for beneficiaries from 1998 to 2008.The dataset was released with de-identified secondary data for public research purposes. All types of personal identification on files connected with the present study were scrambled using surrogate identification numbers to secure patient privacy. The Bureau of National Health Insurance approved the application (NHRI-NHIRD-99182) after reviewing all the required medical documents. Our study received a formal waiver from the institutional review board (IRB) of Chi-Mei Medical Center (No. 1008-002).

### Patient selection and definition

This longitudinal cohort study enrolled 21,051 elderly ESRD patients (≥65 years old) who began maintenance dialysis (hemodialysis or peritoneal dialysis) between January 1, 1999, and December 31, 2005 Maintenance dialysis was defined as undergoing dialysis for more than 90 days. After excluding eight patients who had undergone renal transplantation before beginning dialysis, we were left with a total of 21,043 elderly ESRD dialysis patients to include in our analysis. These patients were followed from the first reported dialysis date to the date of death, renal transplantation, end of dialysis or December 31, 2008. Fifty-four patients received renal transplantation during the follow-up period.

### Ascertaining the demographic and comorbidity variables

We linked to the diagnostic codes through the NHI inpatient and outpatient claims databases. From these databases, we collected demographic, baseline comorbidity, and date of death information. The nCI was used to evaluate the outcomes of these elderly patients [13]. At the start of dialysis, baseline comorbidities were assessed. They included DM, HTN, CHF, coronary artery disease (CAD), cerebrovascular accident (CVA), peripheral vascular disease (PVD), other cardiac disease (pericarditis, endocarditis, myocarditis, other complications of heart disease, heart transplant, heart valve replacement, and cardiac devices), dysrhythmia, chronic obstructive pulmonary disease (COPD), gastrointestinal (GI) bleeding, liver disease, and cancer. The ICD-9-CM codes used to define each condition are shown in Table S1. For more accurate diagnoses of comorbidities, diagnosis for this study was based on whether they fit one of the definitions below: (1) outpatients had to have a diagnosis, designated by ICD-9-CM code, at any time within the year leading up to start of dialysis and had to have received two or more additional diagnoses designated with same code number with the subsequent 12 months; the first and last outpatient visit within 1 year had to be >30 days apart to avoid accidental inclusion of miscoded patients; and (2) inpatients had to have received a diagnosis, designated by ICD-9-CM code, at least one time within the year leading up to start of dialysis.

### Calculating nCI score and CCI score for survival analyses

As in Liu et al. [13], we examined these comorbid conditions in our study population with a Cox proportional hazards model for survival analysis. The eleven comorbid conditions were DM, CAD, CVA, PVD, other cardiac disease, dysrhythmia, COPD, GI bleeding, liver disease, cancer and CHF. We calculated the comorbidity (nCI) score using the following comorbidity-related weight assignments: a weight of 1 assigned to DM and CAD; 2 to CVA, PVD, other cardiac diseases, dysrhythmia, COPD, GI bleeding, liver disease, and cancer; and 3 to CHF (Table 1). The whole sample of elderly patients were divided into four groups base on their nCI scores (intervals ≤3, 4–6, 7–9, ≥10). In these four groups, cumulative survival rate and life expectancy after the initiation of dialysis were further analyzed by age.

**Table 1:** Weighting score for comorbid conditions.

Comorbid conditions	Weighing score
Diabetic Mellitus	1
Congestive Heart Failure	3
Coronary Artery Disease	1
Cerebrovascular Disease	2
Peripheral Vascular Disease	2
Other Cardiac	2
Dysrhythmia	2
Chronic Obstructive Pulmonary Disease	2
Gastrointestinal Bleeding	2
Liver Disease	2
Cancer	2

For comparison, we further collected data on the seventeen comorbid conditions considered for the CCI [15]. We excluded the diagnosis codes of renal disease because all patients had renal disease. Using these data, we calculated the CCI scores in the same elderly dialysis population.

### Statistical analyses

The data were analyzed using the Statistical Package for the Social Sciences for Windows 17.0 (SPSS Inc., Chicago, IL, USA). Baseline characteristics of these patients were compared using χ^2^ test. Overall patient survival was described using the Kaplan-Meier method based on these 4 groups with different comorbidity scores. Relative risks and 95% confidence intervals (CIs) were derived from Cox proportional hazard models. Study subjects were censored if they stop dialysis or were alive until December 31, 2008. The primary outcome (event) was death from any cause. The predictor was the nCI score based on 11 comorbid conditions. Life expectancy was estimated using the life-table method. The remaining expected years of life after the initiation of dialysis was calculated based on each patient's age and nCI score groups. Significance was set at *P*<0.05. In addition, we performed Cox regression analyses in the cohort in which mortality was dependent variable and nCI score and CCI scores were independent variables. From these analyses, the c statistic (equivalent to area under a receiver operating characteristic curve) was used to assess the ability of scores to discriminate between patients who did or did not survive.

## Results

### Demographic characteristics of the patients in different age groups

**Figure 1:**
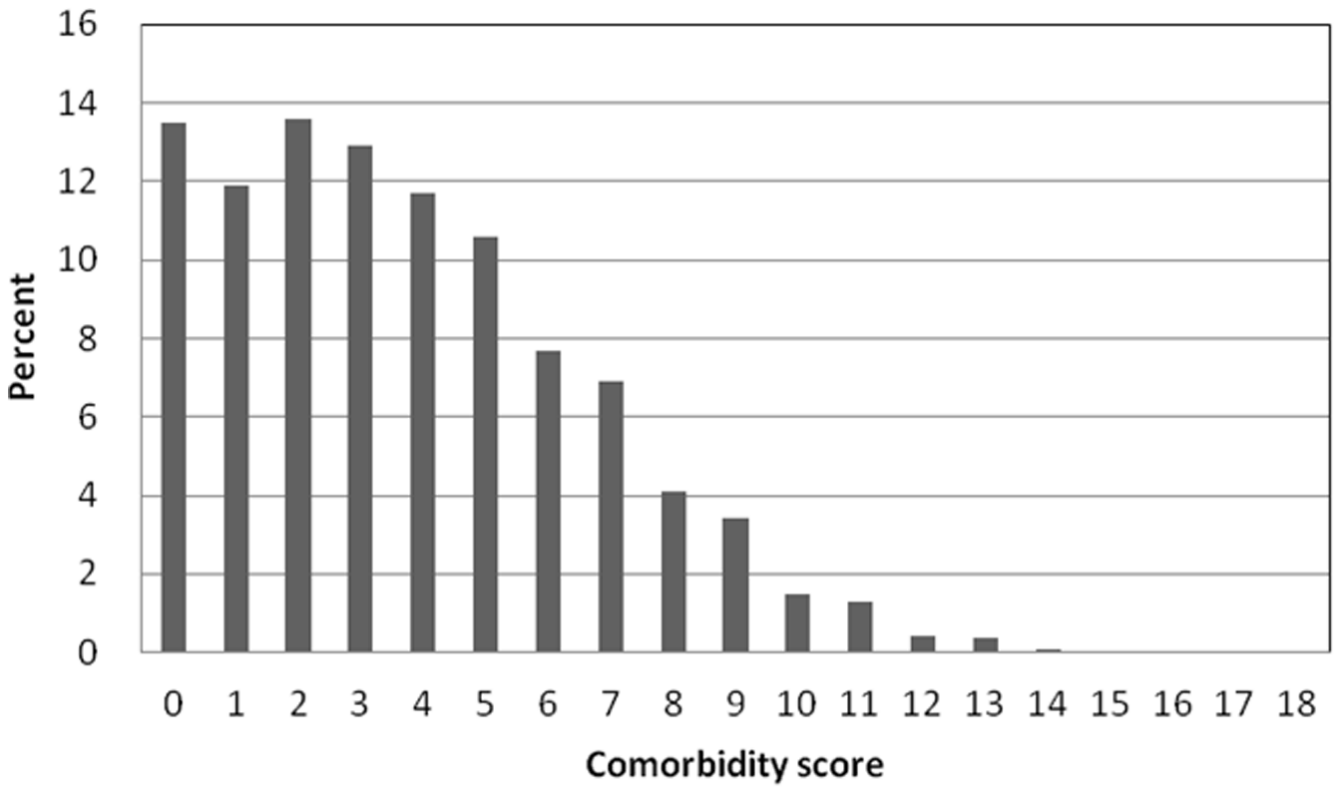
Distribution of elderly dialysis patients by comorbidity score.

This study enrolled a total 21,043 elderly dialysis patients, with 55% (n = 11,517) were women, 96% (n = 20,284) were hemodialysis patients, and 52% (n = 10,915) had diabetes patients (Table 2). The mean duration of follow-up was 3.25 years (median: 1.56 years; interquartile range: 4.82 years). These patients were divided into five groups according to their age (65∼69, 70∼74, 75∼79, 80∼84, ≥85). There were significant differences in gender and nCI scores (≤3, 4–6, 7–9, ≥10) among the 5 age groups (*P*<0.001) as well as a significant difference in the number of patients with and without each of the eleven comorbidities, except peripheral vascular disease (*P*<0.05) (Table 2). Older patients tended have higher nCI score than younger ones. Patients with an nCI score ≥10 had a high prevalence of DM, CHF, CAD, and UGI. The ≤3, 4–6, 7–9, ≥10 nCI score groups had 10913, 6317, 3045, and 768 patients, respectively. Almost fifty-two percent (51.9%) of the patients belonged the group with the lowest nCI score (≤3) group (Figure 1).

**Table 2:** Demographic characteristics stratified by different age group.

	Age 65–69		Age 70–74		Age 75–79		Age 80–84		Age ≥85		P-value
	n	(%)	n	(%)	n	(%)	n	(%)	n	(%)	
Total											
Gender											<0.001
Female	3963	(34.4)	3461	(30.1)	2396	(20.8)	1252	(10.9)	445	(3.9)	
Male	3061	(32.1)	2945	(30.9)	2172	(22.8)	972	(10.2)	376	(3.9)	
New Comorbidity index (nCI)											<0.001
Score ≤3	3854	(35.3)	3356	(30.8)	2295	(21)	1028	(9.4)	380	(3.5)	
Score 4–6	2079	(32.9)	1907	(30.2)	1362	(21.6)	716	(11.3)	235	(4)	
Score 7–9	898	(29.5)	912	(30)	721	(23.7)	369	(12.1)	145	(4.8)	
Score ≥10	193	(25.1)	231	(30.1)	190	(24.7)	111	(14.5)	43	(5.6)	
Initial dialysis modality											0.207
Hemodialysis	6752	(33.3)	6200	(30.6)	4408	(21.7)	2133	(10.5)	791	(3.9)	
Peritoneal dialysis	272	(35.8)	206	(27.1)	160	(21.1)	91	(12)	30	(4)	
Baseline comorbidity											
Diabetes Mellitus											<0.001
No	2904	(28.7)	2949	(29.1)	2414	(23.8)	1318	(13)	543	(5.4)	
Yes	4120	(37.7)	3457	(31.7)	2154	(19.7)	906	(8.3)	278	(2.5)	
Congestive Heart Failure											<0.001
No	5040	(34.5)	4492	(30.8)	3091	(21.2)	1452	(9.9)	518	(3.5)	
Yes	1984	(30.8)	1914	(29.7)	1477	(22.9)	772	(12)	303	(4.7)	
Coronary Artery Disease											<0.001
No	5007	(34.4)	4413	(30.4)	3045	(20.9)	1514	(10.4)	559	(3.8)	
Yes	2017	(31)	1993	(30.6)	1523	(23.4)	710	(10.9)	262	(4)	
Cerebrovascular Disease											<0.001
No	5966	(33.8)	5351	(30.4)	3831	(21.7)	1804	(10.2)	673	(3.8)	
Yes	1058	(31)	1055	(30.9)	737	(21.6)	420	(12.3)	148	(4.3)	
Peripheral Vascular Disease											0.485
No	6650	(33.5)	6050	(30.4)	4315	(21.7)	2084	(10.5)	771	(3.9)	
Yes	374	(31.9)	356	(30.3)	253	(21.6)	140	(11.9)	50	(4.3)	
Other Cardiac											<0.001
No	6382	(33.9)	5740	(30.5)	4070	(21.6)	1940	(10.3)	713	(3.8)	
Yes	642	(29.2)	666	(30.3)	498	(22.7)	284	(12.9)	108	(4.9)	
Dysrhythmia											<0.001
No	6506	(34.5)	5760	(30.5)	4018	(21.3)	1892	(10)	683	(3.6)	
Yes	518	(23.7)	646	(29.6)	550	(25.2)	332	(15.2)	138	(6.3)	
Chronic Obstructive Pulmonary Disease											<0.001
No	6258	(34.8)	5505	(30.6)	3771	(21)	1791	(10)	641	(3.6)	
Yes	766	(24.9)	901	(29.3)	797	(25.9)	433	(14.1)	180	(5.8)	
Gastrointestinal Bleeding											<0.001
No	5190	(33.9)	4705	(30.8)	3250	(21.3)	1566	(10.2)	578	(3.8)	
Yes	1834	(31.9)	1701	(29.6)	1318	(22.9)	658	(11.4)	243	(4.2)	
Liver Disease											0.003
No	6404	(33)	5909	(30.5)	4239	(21.9)	2062	(10.6)	771	(4)	
Yes	620	(37.4)	497	(30)	329	(19.8)	162	(9.8)	50	(3)	
Cancer											0.023
No	6484	(33.6)	5885	(30.5)	4153	(21.5)	2014	(10.4)	756	(3.9)	
Yes	540	(30.8)	521	(29.8)	415	(23.7)	210	(12)	65	(3.7)	

### Examining possible risk factors (the 11 comorbidity variables of nCI) for survival

A Cox proportional hazards model was used to analyze the possible risk factors of mortality in all patients. All eleven comorbidity conditions were significantly associated with mortality (Table 3). Being male rather than female had a hazard ratio (HR) of 1.137 (95% CI: 1.095–1.180), being older rather than younger (vs. 65–69 years) have a HR of 1.268 at 70–74 years, 1.602 at 75–79 years, 2.173 at 80–84 years, 3.057 at ≥85 years, and undergoing hemodialysis rather than peritoneal dialysis had a HR of 1.368 (95% CI: 1.243–1.506).

**Table 3:** Cox model for all-cause mortality among elderly dialysis patients.

Covariate	Relative Risk (95% CI)	*p*-value
Gender (Male vs. Female)	1.137 (1.095–1.180)	<0.001
Age at initiation of dialysis		
65–69 (years)	1	
70–74	1.268 (1.209–1.330)	<0.001
75–79	1.602 (1.522–1.686)	<0.001
80–84	2.173 (2.040–2.314)	<0.001
≥85	3.057 (2.792–3.348)	<0.001
Dialysis modality (HD vs. PD)	1.368 (1.243–1.506)	<0.001
Diabetic Mellitus (DM) (yes vs. no)	1.503 (1.448–1.560)	<0.001
Congestive Heart Failure (CHF) (yes vs. no)	1.482 (1.425–1.541)	<0.001
Coronary Artery Disease (CAD) (yes vs. no)	1.305 (1.255–1.357)	<0.001
Cerebrovascular Disease (yes vs. no)	1.452 (1.384–1.524)	<0.001
Peripheral Vascular Disease (yes vs. no)	1.175 (1.086–1.270)	<0.001
Other Cardiac (yes vs. no)	1.181 (1.113–1.252)	<0.001
Dysrhythmia (yes vs. no)	1.338 (1.263–1.418)	<0.001
Chronic Obstructive Pulmonary Disease (yes vs. no)	1.349 (1.283–1.419)	<0.001
Gastrointestinal (UGI) Bleeding (yes vs. no)	1.192 (1.144–1.241)	<0.001
Liver Disease (yes vs. no)	1.256 (1.176–1.341)	<0.001
Cancer (yes vs. no)	1.315 (1.233–1.403)	<0.001

HD, hemodialysis; PD, peritoneal dialysis.

### Cumulative survival rate

**Figure 2:**
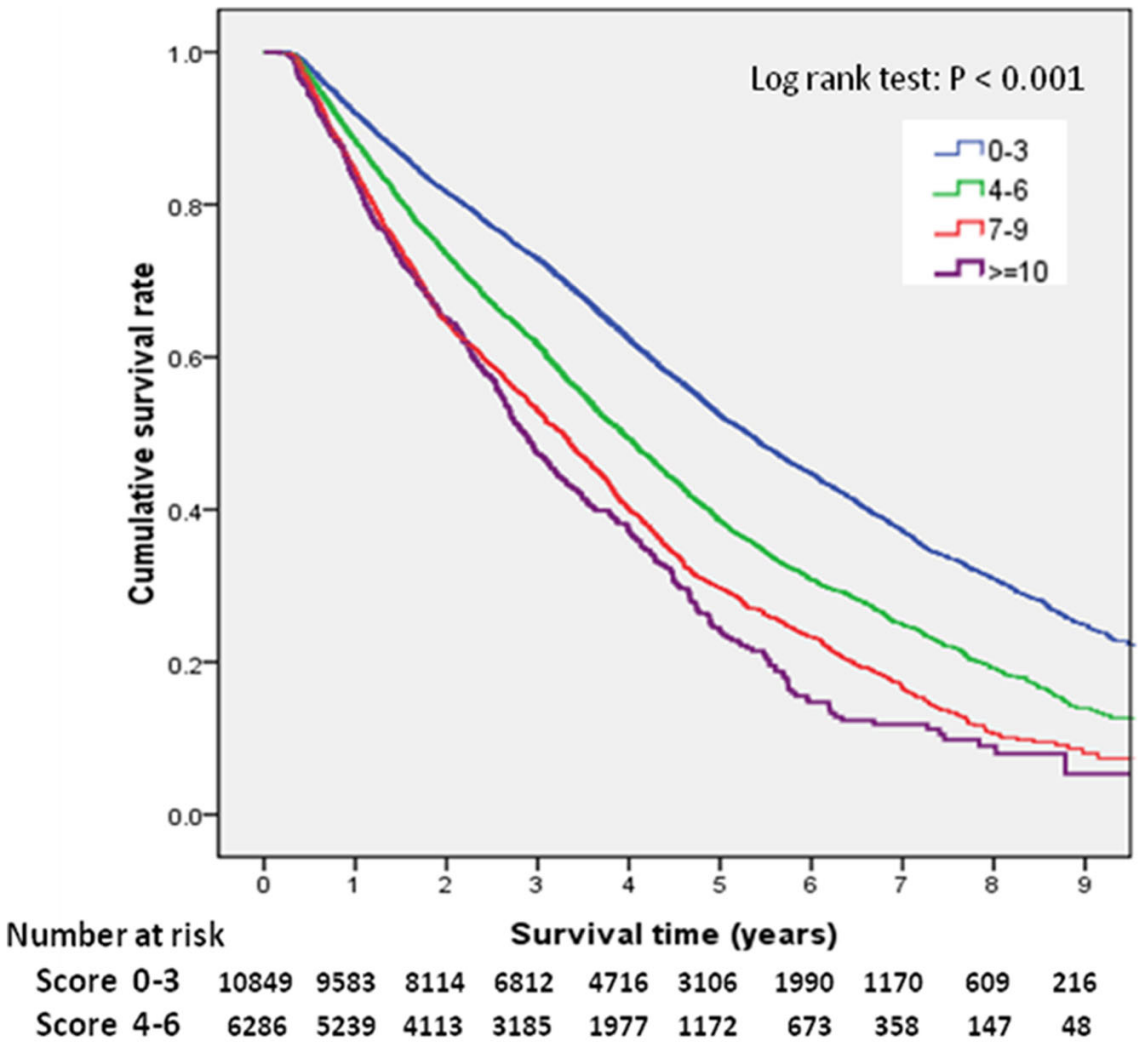
Survival curves for elderly patient groups with comorbidity scores(≤3, 4–6, 7–9, and ≥10). Further analysis of only the third (7–9) and fourth (≥10) groups was performed, and a significant difference was found (log-rank: P = 0.017).

During the 10-years follow-up period, 11272 (53.55%) patients died. The crude all-cause mortality rate was 15.5/100 patient-years (Figure 2). Mean follow-up time alive on dialysis was 4.99 years (95% CI: 4.93–5.04 years). The cumulative survival rate of the lowest score group (≤3) was 88.3% at one year, 28.6% at five years, and 2.0% at nine years. The cumulative survival rate of the highest score group (≥10) was 75.0% at one year, 9.3% at five years, and 0.1% at nine years. The differences in survival between these four groups were significant (log-rank: *P*<0.001).

Patients in lower nCI score groups had a better survival rate. The relative risks for the 4–6, 7–9, and ≥10 groups relative to ≤3 group were 1.533, 2.117, and 3.138, respectively. These results show the nCI score to be an important predictor for survival in this elderly dialysis population.

### Comorbidity-adjusted life expectancy after beginning dialysis

**Figure 3:**
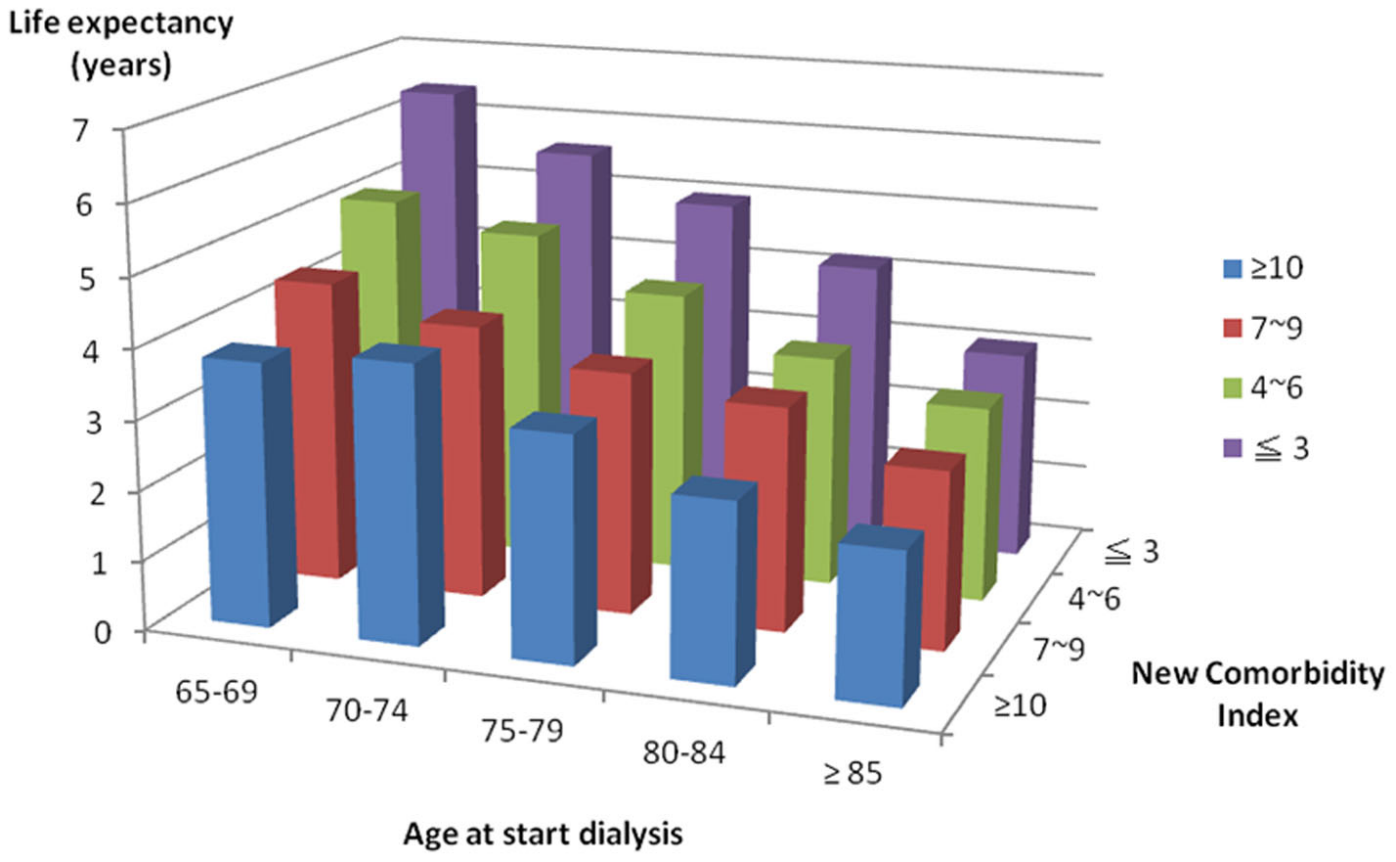
Estimated life expectancy after the initiation of dialysis in these elderly patients by study period.

Estimates of mean life expectancy after beginning dialysis ranged from 2.87 to 5.57 years, depending on the patient's age at the start of dialysis. In all age groups, a significantly higher life expectancy was found in lower nCI score groups (Table 4; Figure 3).

**Table 4:** Estimated life expectancy after dialysis initiation by study period.

Age at start		new Comorbidity Index (nCI)			
of dialysis	Total	≤3	4–6	7–9	≥10
65–69	5.757 (2.664–5.849)	6.415 (6.293–6.536)	5.144 (4.980–5.308)	4.389 (4.148–4.629)	3.790 (3.373–4.206)
70–74	5.098 (5.004–5.192)	5.603 (5.474–5.732)	4.800 (4.625–4.975)	3.951 (3.729–4.173)	3.981 (3.530–4.431)
75–79	4.427 (4.323–4.531)	4.968 (4.817–5.119)	4.079 (3.899–4.259)	3.484 (3.270–3.699)	3.222 (2.840–3.604)
80–84	3.696 (3.555–3.837)	4.178 (3.961–4.396)	3.343 (3.115–3.571)	3.214 (2.891–3.537)	2.552 (2.160–2.943)
≥85	2.871 (2.684–3.058)	3.045 (2.763–3.327)	2.823 (2.498–3.147)	2.549 (2.192–2.905)	2.132 (1.687–2.577)

## Discussion

When an elderly person, especially the very old (≥85 years old), faces the possibility of dialysis, the nephrologist often has difficulties making treatment decisions as he or she must consider many aspects regarding the patient's underlying condition and probable outcome [16], [17]. These decisions must also be faced by both the patients and their families, as they share in the decision making. Mortality in elderly patients has been closely correlated with many comorbidities independent of age [18], [19]. Therefore, some scoring systems have been developed to help the physician assess whether a dialysis patient will live long enough to benefit from the therapy and have their life span prolonged [10]–[12], [20]. However, most studies have not focused on elderly dialysis patients, some of whom may need several clinical assessments and laboratory datasets just to predict short-term (6–12 months) survival [20], [21]. For all of these scoring systems, age has always been a strong independent predictive factor. Liu et al. modified the CCI without including the age factor and developed the nCI to analyze outcomes for dialysis patients [13]. The nCI was found to have a good predictive value and was reliably reproducible in the large USRDS database dialysis population. Our study used the nCI and Taiwan's NHIRD dialysis population to try to develop a relatively easy approach to predict the outcomes of elderly patients after beginning dialysis. We found that the patients in the highest score group had the highest mortality risk, and that after 5 years of dialysis, the survival rate of patients in the lowest score group was three times better than that of patients in the highest score group. In addition, this study compared the nCI with the Charlson comorbidity index using the predictive ability statistic (c-statistic for time-to-event data). The c-statistics used to measure to what extent predicted values from the model. This study found the c-statistics value of nCI score to be 0.90782 (95% CI: 0.89685–0.91823), while that of Charlson comorbidity index (CCI) score to be 0.90035 (95% CI: 0.88901–0.91115). Therefore, the two models were found to have virtually identical performance. However, when applying the nCI, a physician would not need to consider as many comorbid condition variables as CCI, which would make it simpler and more convenient to use in clinical practice.

Age was not included in the comorbidity index of the nCI because it is difficult to “sum” the effects of age and comorbid conditions while assigning definite scores [13]. However, we stratified the four score groups by age and found a good correlation between age group and survival rate. During 1999–2000 period, the estimated life expectancy of the general population in Taiwan was 76.5 years (male: 73.8 years; female: 79.6 years) [22], and most elderly patients (especially those ≥70 years) with lower nCI scores lived almost that long. In addition, the number of comorbid conditions may also reveal an inverse relationship with the quality of life [23]. Therefore, we can expect that elderly dialysis patients with lower nCI scores to live with a quality of life almost as good as that of the elderly in the general population. One Canadian study found the mean life expectancy of 75- to 80-year-old dialysis patients to be 3.2 years [24]. This is only slightly longer life than the expectancy in our study population, especially for patients with lower nCI scores.

In contrast, our study found patients with higher nCI scores to have a shorter life expectancy. Similarly, one recent study found that dialysis may not be beneficial for the survival of patients over 75 years old who have multiple comorbidities and cardiac ischemia [16]. Although dialysis definitely provides a life-sustaining therapy and extends patients' lives, it may also aggravate or prolong a patient's suffering for the remainder of his or her life, and even extend the dying process. Elderly dialysis patients may suffer from a substantial and sustained deterioration of functional status after beginning dialysis, especially if they reside in nursing home [25]. Because elderly patients generally have more comorbid conditions than younger patients, they may suffer more in daily life from the related complications. For example, PVD-related amputation causes not only severe functional impairment, but also presents a huge health burden in many countries [26]. In such a population, we might consider renal palliative care over dialysis for elderly patients >75 years old who have more comorbidities (i.e., higher nCI scores), especially those with poor daily living status [27].

According to the Renal Physicians Association and the American Society of Nephrology (RPA/ASN) guidelines [28], pre-ESRD patients and their families should receive clear information about their prognosis and all treatment options before shared decision making about whether or not to begin dialysis. However, these guidelines do not provide specific reliable means for making an overall prognosis estimate in octogenarians [29]. Several studies also found that patients with poor outcomes still expected their physicians to explain their prognosis in detail [30]. Our study provided quantitative estimates of life expectancy in different nCI score groups and age groups. These findings may support the physician for satisfactory explanations to elderly patients about to begin dialysis and also be used to encourage patients without many comorbid conditions (low nCI scores).

This study found elderly women comprise over half of this dialysis population in Taiwan. This preponderance of women in Taiwan dialysis population has been also found in another published study using NHIRD [31]. This study also found 51.5% of the patients to belong to the lowest nCI score group. One possible explanation for this preponderance is that elderly patients with chronic kidney disease may have high rates of multiple comorbid conditions [32] and, therefore, may die at a younger age because of the related complications of their underlying chronic diseases (such as DM, CAD, and CHF). They would be expected to have a relatively shorter life span without facing the choice of dialysis. In addition, there is the possibility of selection bias, since elderly patients with ESRD and multiple comorbid conditions might be more likely to be treated with palliative therapy because of their short life expectancy and would, thus, not live as long as those undergoing dialysis [18]. Nevertheless, we found significant differences in survival between the four nCI score groups. Because many of these elderly patients were in the lowest nCI score group with relatively few comorbid extrarenal conditions at the beginning of dialysis, age alone should not be the only or the most important consideration for decision-making. More aggressive dialysis therapy should be considered a preferred choice for patients in the low nCI score group, even for octogenarians [18], [19].

This study has several important limitations. First, there is no definite classification of severity for each comorbid condition and functional status. However, according to the NHI payment provisions, all treatment procedures and medications require an associated diagnostic code. Therefore, while we recorded the diagnostic codes given to each patient, comorbid disease severity should be “clinically evident” based upon the prescribed treatment. Even if we underestimated the prevalence of comorbidities, the nCI system provided a good predictive value. Second, using this billing database, we were unable to consider body mass index, actual blood pressure, specific data on dialysis adequacy, type of vascular access used for HD patients, laboratory data, and medical prescriptions, which may also have affected each patient's survival. We were also unable to identify functional status, an important prognostic factor in elderly dialysis patients. Third, although the nCI seemed to have the identical performance as CCI, we did not to validate the nCI with the categories in an independent population. Finally, it would be better to describe the causes of death for further analyses. Unfortunately, the Taiwan Bureau of National Health Insurance does not make available the cross-link information between it NHI database and “causes of death” database.

## Conclusion

Our study found that nCI, even without the age component, is still a strong predictor of mortality in elderly dialysis patients. Old age alone should not be used as an absolute barrier to treatment when considering the benefits of dialysis in elderly ESRD patients. The elderly dialysis population with lower nCI scores (i.e. fewer comorbidities) might have better survival. Adequate dialysis rather than palliative care should be considered preferentially in this population, especially in patients without obvious functional impairments.

## Supporting Information

Table S1 **ICD-9-CM codes used to identify clinical conditions.**
(DOC)
