# Supplementary material for: The New Comorbidity Index for Predicting Survival in Elderly Dialysis Patients: A Long-Term Population-Based Study
Source: PLoS One. 2013 Aug 6;8(8):e68748. doi: 10.1371/journal.pone.0068748 (PMC3735534; doi:10.1371/journal.pone.0068748)
Supplement: Table S1 — ICD-9-CM codes used to identify clinical conditions. (DOC) [file pone.0068748.s001.doc]

**Table S1. ICD-9-CM codes used to identify clinical conditions**

|  | |
| --- | --- |
| **Conditions** | ***ICD-9-CM*** |
| Diabetes mellitus | 250; 357.2; 362.0X; 366.41 |
| Congestive heart Failure | 398.91; 422; 425; 428; 402.X1; 404.X1; 404.X3 |
| Coronary artery disease | 410- 414 |
| Cerebrovascular accident/TIA | 430-438 |
| Peripheral vascular disease | 440-444; 447; 451-453; 557 |
| Other cardiac | 420-421; 423-424; 429; 785.0-785.3 |
| Chronic obstructive pulmonary disease | 491-494; 496; 510 |
| Gastrointestinal bleeding | 456.0-456.2; 530.7; 531-534; 569.84; 569.85; 578 |
| Liver disease | 570; 571; 572.1; 572.4; 573.1-573.3 |
| Dysrhythmia | 426-427 |
| Cancer | 140-172; 174-208; 230-231; 233-234 |
| ICD-9-CM, *International Classification of Diseases, Ninth Revision, Clinical Modification*; TIA, transient ischemic attack | |
